# Using films as a psychoeducation tool for patients with schizophrenia: a pilot study using a quasi-experimental pre-post design

**DOI:** 10.1186/s12888-015-0481-2

**Published:** 2015-04-30

**Authors:** Christian von Maffei, Frauke Görges, Werner Kissling, Wolfgang Schreiber, Christine Rummel-Kluge

**Affiliations:** Department of Psychiatry and Psychotherapy, University of Leipzig, Semmelweisstr. 10, D-04103 Leipzig, Germany; Forschungszentrum Depression der Stiftung Deutsche Depressionshilfe, Semmelweisstr. 10, 04103 Leipzig, Germany; Department of Psychiatry and Psychotherapy, Technische Universität München, Möhlstr. 26, 81675 München, Germany; Bezirksklinikum Mainkofen, 94469 Deggendorf, Germany

**Keywords:** Schizophrenia, Psychoeducation, Film, Group

## Abstract

**Background:**

Relapses and, subsequently, readmissions are common in patients with schizophrenia. Psychoeducation has been shown to reduce the number and duration of readmissions. Yet, only little more than 20% of psychiatric patients in German speaking countries receive psychoeducation. Among other reasons, costs may be considered too high by hospitals. The objective of the present study was to test the feasibility of a new cost-efficient approach in the psychoeducation of patients with schizophrenia. In this study, films were used to impart knowledge about the illness to inpatients.

**Methods:**

A total of 113 participants were initially included in the study, eleven of which were not included in the final analyses. Six films about the symptoms, diagnosis, causes, warning signs, treatment of schizophrenia and about the influence of family members and friends were shown in a group setting in the presence of nursing staff. All films combined facts, expert opinions, and personal experiences of peers. As the main outcome criterion of this feasibility pilot study, we measured the effects on knowledge. Secondary outcome measures included compliance, insight into illness, side effects, and quality of life. Data were collected directly after the intervention and about half a year afterwards. The number and the duration of readmissions to the hospital were recorded and compared to the number and duration of prior admissions. Patients were also asked to state their subjective opinion about the films. Main data analyses were done using paired *t*-tests and Wilcoxon signed-rank tests. Secondary analyses also involved ANOVAs and ANCOVAs.

**Results:**

One hundred and two inpatients were included in the data analyses. Showing the films in the tested setting was shown to be feasible. Knowledge about schizophrenia (*p* < .001), compliance (*p*s < .01), insight into illness (*p* < .01), and quality of life (*p* < .001) all increased significantly after patients had watched the films and remained stable for at least half a year. A vast majority (84.9%) of the patients found the films to be interesting and informative.

**Conclusions:**

Using films to educate inpatients about schizophrenia is a feasible method that is cost- and time-efficient and well received by the patients.

## Background

Schizophrenia is a disease that often takes a chronic course. Relapses with and without readmission to the hospital are common and often associated with medication non-compliance [1-8]. There are various causes for non-compliance, including informational deficits, consequences of psychopathological symptoms, such as cognitive impairments or poor insight into illness, subjective response to medication, and side effects [9-12].

Relapses come at a high price for both the patient and the health care system. The relationship between non-compliance and increased costs for treatment has become apparent in previous research [6,13,14]. Different approaches have been taken to improve compliance and to thereby reduce the costs of (partial) non-compliance, several of which have been proven to be effective [15]. One promising approach is psychoecucation [16]. Researchers found positive effects of psychoeducative programs on knowledge, compliance, psychopathology, satisfaction with treatment, and global functioning. They were also related to a change of illness concept and to fewer relapses as well as fewer and shorter readmissions to the hospital [17-20].

In a large-scale randomized multicenter study involving 236 inpatients (Psychosis Information Project Study), Pitschel-Walz et al. [21] investigated the long-term effects of a short psychoeducative program on the number and duration of readmissions, compliance (rated by treating physician) and global functioning (GAF) 12 and 24 months after index hospitalization. The program comprised 8 one-hour group sessions, in which patients received information about symptoms, etiology, treatment, and relapse prevention. Close relatives received a similar group intervention. For all the outcome variables, the authors demonstrated positive effects of psychoeducation on the intervention group compared to a control group, who received treatment as usual. An increase in knowledge and an improvement in illness concept were also observed in a 6-month follow-up questionnaire [22]. Despite the great number of studies demonstrating the effectiveness of psychoeducative programs and calls for them to be a part of standard treatment [16], this form of intervention is still not implemented across the board.

According to a study investigating the implementation of psychoeducation for schizophrenia, in 2003, programs were offered by 83% of hospitals in Germany, Austria, and Switzerland. However, overall, only 21% of the patients received psychoeducation. Dropout was high with a rate of 25% [23,24]. Several factors may have contributed to this situation. Some hospitals may still question the effectiveness of such programs, but most of the hospitals simply do not have enough staff to provide a well-prepared weekly psychoeducation program for their patients. And even for those who do, reaching their patients seems to be a difficult task. In some patients, symptoms may be too severe. Others are discharged (with or against medical advice) before they complete the program, and some patients lack the motivation to join or complete the program. While, from the hospital’s and the patient’s point of view, many of these reasons for not offering or participating in psychoeducation are understandable, the costs are high. Rummel-Kluge et al. estimated that up to 150 million Euros could be saved each year by tripling the number of patients receiving psychoeducation [23].

The aim of the pilot study reported here was to investigate the feasibility of a short, time- and cost-efficient tool for psychoeducation, which would also be well-received by patients. To tackle the issue of psychoeducation being too time consuming and expensive, we used a program consisting of six films, in which the main topics concerning schizophrenia and its treatment are covered [17]. The patients’ motivation was addressed by making the films shorter than the usual one-hour program and by presenting facts more vividly by backing them up through expert opinions and by adding stories of other patients’ personal experiences. Previous research showed that peers as counsellors and educators are very well received by psychiatric patients [25-27]. In a quasi-experimental pre-post design, several measures were assessed. As the main outcome variable, we measured the patients’ knowledge about their illness before and after the intervention. Secondary outcome variables included the number and duration of readmissions, compliance, insight into illness, side effects, and quality of life. Most of these measures were assessed before, immediately after the intervention, and approximately half a year after the intervention. Compliance was measured before and half a year after the intervention. Readmission data were taken from the hospital data base. In addition, patients were asked to state their personal opinion on the content and on the interestingness of the films. Both objective and subjective measures were used to gain insight into the feasibility of this new approach.

## Methods

### Intervention

Six films of about 17 minutes in length each were used for this psychoeducative program. Originally, the films were part of a larger psychoeducation program (Alliance Psychoeducation Program [17]). The script was written by two of the authors of the present study and the films were produced by a professional company. The films were very well received by patients who participated in the evaluation of the Alliance Psychoeducation Program, leading up to the idea to use the films standalone. They included objective information about schizophrenia given by a presenter. Objective information was accompanied by clips in which patients gave statements about their personal experiences. Information about the treatment of schizophrenia was given by experts. Films were presented continuously in a fixed order (1. symptoms of schizophrenia, 2. diagnosis of schizophrenia and dealing with schizophrenia, 3. causes of schizophrenia, 4. medication effect and side effects, 5. warning signs before relapse, 6. influence of family members and friends) with each of the cycles lasting two weeks. On average, patients watched three films per week. They could enter the program at any point, starting with the film that was presented next. A member of the nursing staff was always present, but did not actively interfere with the program. However, the nurse was allowed to respond to questions. At the end of each session, patients were asked to give a short, written evaluation of the film. A session lasted for about 35 minutes, including the evaluation.

Patients received standard psychiatric care with psychopharmacological treatment. Written informed consent from each patient and institutional review board approval were obtained (Ethics Committee of the Faculty of Medicine, Technische Universität München, Germany).

### Participants

A total of 113 patients from a psychiatric hospital in Mainkofen, Germany were initially included in the study. Patients were included if they met the following criteria: a) aged 18–65, b) diagnosis of schizophrenia or schizoaffective disorder according to ICD-10, c) according to the clinical decision of the treating physician, psychotic symptoms had decreased enough to be able to follow psychoeducative content and to fill out questionnaires, d) no signs of organic brain damage or intellectual disability, e) adequate knowledge of the German language, f) adequate eye sight, and g) estimated duration of stay of at least two weeks. Participants were included in the data analyses if they had watched at least five of the six films. Eleven patients did not meet the criteria (e.g. due to change in diagnosis) and had to be excluded from data analyses.

### Assessment

There were two main measuring points for data assessment: immediately before the intervention (T0) and immediately after the intervention (T1; on average 15 days after the first assessment). In addition, it was possible to obtain data from some patients and their physicians a few months after the intervention to assess the stability of the effects (T2; for patients on average 150 days and for doctors on average 163 days after the first assessment).

### Self-assessment

The Knowledge of Illness about Schizophrenia Questionnaire [28] was used to assess a potential increase in knowledge. It is an instrument for assessing knowledge of patients with schizophrenia about symptoms, causes, treatment, and warning signs. It comprises 21 questions with 107 single items. The maximum score is 70 points. The questionnaire is reliable (reliability coefficient α = .91) [19,29].

In order to assess the adherence to medication, the Medication Adherence Rating Scale (MARS) was used. The MARS comprises ten items. The score ranges from 0 to 10 points with 10 points indicating more compliance. The MARS shows high internal consistency with a Cronbach’s alpha of .75 [30].

In addition, single items were used to assess how well the patient felt informed about schizophrenia (4-point scale: 1 – very good, 2 – good, 3 – moderate, 4 – poor), perceived quality of life (5-point scale: 1 – very poor, 2 – poor, 3 – average, 4 – good, 5 – very good), contentment with health (5-point scale: 1 – very discontent, 2 – discontent, 3 – neither content nor discontent, 4 – content, 5 – very content), and the subjective rating of the strength of medication side effects (Visual Analogue Scale with 0 indicating strong side effects and 100 indicating no side effects).

### External assessment

All external assessments for T0 and T1 were done by nurses from the hospital. External assessments for T2 were done by the psychiatrist/neurologist or general practitioner, from whom the patient received outpatient treatment at the time. The physicians evaluating T2 measures were blind to the nurses’ assessment at T0 and T1. Compliance was only measured at T0 and T2, not at T1.

Compliance was rated on a 4-point scale that was exactly operationalized (1 – very good, 2 – good, 3 – moderate, 4 – poor; see Pitschel-Walz et al. [21] for exact operationalization) and insight into the illness was rated on a 3-point scale (1 - insightful, 2 – somewhat insightful, 3 – not insightful; fourth alternative: not relevant). This item was taken from the Scale to Assess Unawareness of Mental Disorder. Inter-rater ICC for the item was .89 [31].

Frequency and duration of (re-)admissions to the hospital were obtained from the hospital data base for 70 weeks prior to the index admission and for 70 weeks after index discharge.

Immediately after watching a film, participants were presented with three statements and asked to check a box if they agreed with them. The statements were the following: “It was interesting and informative.”, “I already knew most of the content.”, “I was too troubled by this”. In addition, they were asked to write down what they had liked most and what had helped them most.

### Statistical analysis

Descriptive methods were used for statistical analyses of sociodemographic and feedback data from participants. For Visual Analogue Scales and summed scores, such as the Knowledge of Illness score and the MARS score as well as the subjective strength of side effects, equidistance and normality [32] were assumed. These comparisons were made using paired *t*-tests. For our primary outcome measure, the 95% confidence interval of the difference was analyzed. Comparisons involving all other data were made using the Wilcoxon signed-rank test. Exploratory analyses, which are reported in the discussion, were made using ANOVAs to test for an interaction of prior treatment with the increase in knowledge and ANCOVAs to test for the influence of perceived side effects on compliance. All calculations were done with PASW statistics, versions 18.0.0 and 20.0.0.2.

## Results

### Reception of films

Overall, the films were well received by the patients. On average, 84.9% of the participants thought them to be interesting and informative. To an average of 82.3%, most of the content of the films was new. Only 7.5% thought that the films were too upsetting to watch, while 92.5% did not think so.

### Pre-post comparisons

Sociodemographic data are presented in Table 1. Means, standard deviations, and sample sizes for each dependent measure are given in Table 2.

**Table 1:** **Sociodemographic data of participants, n = 102**

**Characteristic**	**M**	**SD**
Age (years)	35.1	9.0
Duration of Stay (days)	57.4	26.6
Duration of Illness (years)	11.1	10.6
Number of prior hospitalizations*	3.8	4.0
	**n**	**%**
Gender		
Male	69	67.6
Female	33	32.4
Diagnosis		
Schizophrenia	86	84.3
Schizoaffective Disorder	16	15.7
Pharmacological Treatment		
Atypical Antipsychotic	74	72.5
Typical Antipsychotic	15	14.7
Both	13	12.7
Prior participation in a psychoeducation program		
Yes	16	15.7
No	85	83.3
n/a	1	1
Employment status		
Unemployed	26	25.5
In training	6	5.9
Competitive employment	23	22.6
Non-competitive employment	6	5.9
retired	34	33.3
n/a	7	6.8
Living status		
Independently	30	29.4
With parents/siblings/relatives	40	39.2
With partner/children	25	24.5
In a rehabilitation facility	7	6.9

*Information was available from 96 patients.

**Table 2:** **Means and standard deviations (in Parentheses) for T0 and T1, as well as sample sizes for each measure**

**Measure**	**T0**	**T1**	n*
**Knowledge of Illness about schizophrenia questionnaire**	**41.1 (14.2)**	**48.4 (14.2)**	**92**
**MARS**	**7.2 (2.0)**	**7.9 (1.8)**	**73**
**Insight into Illness**	**1.6 (0.7)**	**1.4 (0.6)**	**91**
**Feeling of being informed about schizophrenia**	**2.7 (0.9)**	**1.8 (0.7)**	**94**
Subjective strength of side effects	65.2 (28.1)	65.8 (25.8)	90
**Perceived quality of life**	**3.0 (1.0)**	**3.5 (0.9)**	**92**
**Contentment with health**	**3.0 (1.2)**	**3.5 (1.0)**	**91**
	**Pre**	**Post**	N*
**Compliance**	**2.2 (1.0)**	**1.7 (1.0)**	**54**
Frequency of (re-)admission	0.5 (1.0)	0.6 (0.9)	101
Duration of (re-)admission	22.8 (49.4)	26.3 (42.2)	101

Significant results are indicated by bold text.

*Data were not available from every patient for each outcome measure. As many data points as possible were included in each analysis, causing the sample size to differ between outcome measures.

### Self-assessment

There was a significant increase of 7.3 points in the knowledge of illness score, *t*(91) = −9.87, *p* < .001. The 95% CI [5.8, 8.8] of the difference between the two means did not include the value “0” and therefore corroborates the finding of a significant increase in our primary outcome measure.

Patients also scored higher in the MARS, *t*(72) = −3.90, *p* < .001, after they had participated in the psychoeducation program. They felt more informed about schizophrenia, *Z* = −6.52, *p* < .001, rated quality of life higher, *Z* = −4.70, *p* < .001, and felt more content with their health, *Z* = −4.21, *p* < .001. The subjective rating of the strength of medication side effects did not decrease *t* < 1.

### External assessment

There was a significant increase in the rating of insight into illness, *Z* = −2.90, *p* < .01. However, neither frequency nor duration of (re-)admissions to the hospital changed significantly after the intervention, all *p*s > .140.

### Long-term effects

Data for T2 are available from only about half of the patients. Thus, they were analyzed separately and should be interpreted carefully.

Compliance, as rated by nurses and doctors, increased significantly, *Z* = −2.62, *p* < .01. The knowledge of illness score was still elevated at T2 when compared to T0, *t*(52) = −4.35, *p* < .001 and did not differ from T1, *t*(52) = 0.88, *p* = .384 (n = 53). The same pattern was found for the MARS score, T0-T2: *t*(42) = −2.14, *p* = .038 and *t*(42) = 0.15, *p* = .883 respectively (n = 43), perceived quality of life, *Z* = −2.27, *p* < .05 and *Z* = −0.66, *p* = .509 respectively (n = 51), contentment with health, *Z* = −1.99, *p* < .05 and *Z* = −0.62, *p* = .533 respectively (n = 51), and insight into illness, *Z* = −2.32, *p* < .05 and *Z* = −0.57, *p* = .567 respectively (n = 49).

Patients still felt more informed about schizophrenia at T2 when compared to T0, *Z* = −4.83, *p* < .001. There was, however, a small decrease from T1 to T2, *Z* = -1.96, *p* < .05 (n = 54). Again, there were no effects for the subjective rating of the strength of medication side effects, *t* < 1 (n = 54).

## Discussion

The present study aimed at investigating the feasibility of a new approach for psychoeducation that is more time- and more cost-efficient compared to a standard psychoeducation program, while integrating vital information about schizophrenia given by professionals with the personal experience of peers, who patients can relate to. The intervention proved to be effective in several measures.

### Knowledge about schizophrenia increased

Patients were more informed about their illness at the end of the psychoeducation program and seemed to still remember this information after a few months had passed. Subjectively, patients also felt more informed about schizophrenia immediately after the intervention, but seemed to feel somewhat less confident after a few months. This effect might be reduced if patients had the opportunity to visit booster sessions or if the films were made available for private use, giving further access to information and to emotional support after being discharged. The two-month period directly following a hospitalization is especially important for adherence as the readmission rate is particularly high here [33]. Being able to watch the films at home may also aid those patients, who were not able to complete the program at the hospital due to early discharge and, as a desirable side effect, this also raises the likelihood of relatives watching the films. The importance of educating relatives has been shown in many studies, however, it is still not being done thoroughly in severe mental illnesses, where stigma and social avoidance remain a serious problem [16,34-37].

### Compliance and insight into illness increased

Compliance was also higher after the intervention as indicated by the self-assessment (MARS) and external assessment (rated by nurses and doctors). The increase in compliance seemed to be long-term. Alongside compliance, insight into illness, which might be a crucial prerequisite, also increased. Unfortunately, the number of (re-)admissions did not decrease in the first 70 weeks after index discharge. However, schizophrenia often takes a chronic course making readmissions a necessity to protect the patients from the consequences of their disease. Thus, readmissions, especially if they happen voluntarily and early during a relapse, can be considered a success. This notion is supported by the increase in the quality of life ratings. Although the patients spent about as much time in the hospitals as they had done before the index treatment, they were more content with their health and perceived a higher quality of life.

However, compliance may have increased because the medication started to work, thereby also reducing the consequences of psychopathological symptoms, such as impaired cognitive functions and impaired insight into illness. However, patients were only included in the study if they were able to follow the content of the films and to fill out questionnaires, implying the ability to be compliant. In addition, the Clinical Global Impression Severity score did not change significantly between T0 (*M* = 5.9, *SD* = 0.7) and T1 (*M* = 5.8, *SD* = 0.8), *p* = .354. The patients’ attitude towards their medication may have been better due to a better adjustment of that medication at the end of treatment, causing fewer side effects. However, the perceived strength of side effects did not differ between any of the times of measurement. In an ANCOVA with the perceived strength of side effects at T1 as covariate, the covariate only influenced the MARS score at a trend level, *F*(1,69) = 3.25, *p* = .076, *MSE* = 6.14, and did not influence compliance or insight into illness at all, *F* < 1 *and F*(1,84) = 1.93, *p* = .168, *MSE* = 0.58 respectively. For the MARS score, the difference between T0 and T1 remained significant after controlling for the perceived strength of side effects, *F*(1,70) = 13.53, *p* < .001, *MSE* = 1.10. Future studies, however, should also evaluate cognition, which can be an important factor in knowledge acquisition.

### Films were well accepted

There is evidence that insight into a severe mental illness is also associated with negative implications such as self-stigma, shame or lower quality of life [38-40]. The films might have had a similar effect on the patients in the present study. However, our results suggest that any negative effect, if present at all, could not have lasted long. We would have expected more patients to find the films upsetting, which was not the case. Also, perceived quality of life increased in the present study. Any negative effect of gaining insight through the films may have been counteracted by the combination of providing facts and personal experiences of successfully treated patients in the films.

### First and multiple episode patients benefit from the intervention

Both, first time patients (n = 63; *t*(62) = −8.74, *p* < .001) and others (n = 29, *t*(28) = −4.84, *p* < .001) showed a significant increase in knowledge. The interaction of prior treatment with time of measurement for the knowledge of illness score did not exceed a trend level, *F*(1,90) = 3.15, *p* = .079, *MSE* = 24.84). Importantly, this result indicates that psychoeducation is an effective tool not only during a patient’s first episode, but also after one or more relapses.

### Limitations

There are certain limitations to this study, the most obvious one being the use of a quasi-experimental design. Due to the lack of a control group that did not receive the same treatment as the intervention group, we cannot rule out beyond any doubt the possibility that our results were in fact caused by something other than the intervention. For instance, it seems possible that treatment as usual alone may have caused the effects reported above. Patients may have learned about schizophrenia from the hospital staff or from other patients. While this is probably true to some extent, knowledge would have been acquired in a less systematic way, causing variance to be bigger and effects to be smaller. In a previous study, Pitschel-Walz et al. [22] compared the increase in knowledge between an intervention group that participated in a psychoeducative group program and a control group that received treatment as usual. An increased score in the Knowledge of Illness about Schizophrenia Questionnaire was observed for the intervention group only, not for the control group. Taking these results into consideration, it seems highly unlikely that treatment as usual was the cause of the effects observed in the present study. In addition, we would have expected first time patients to have a much higher increase in knowledge than patients who had been treated for schizophrenia in a hospital before. This was not the case. Furthermore, we cannot rule out the possibility that choosing a mixed diagnoses sample may have had an influence on the results. The program was originally planned for patients with a diagnosis of schizophrenia. Patients who suffer from schizoaffective disorder differ in some aspects from patients with schizophrenia and may have responded differently to the content of the films. Unfortunately, the sample of patients with schizoaffective disorder was too small to be analyzed separately. Future research needs to determine the effect of the films on this group of patients.

## Conclusions

Using films to educate patients about schizophrenia and schizoaffective disorder is a cost-saving and time-efficient approach, which was well received by the vast majority of our patients. We were able to show positive effects on knowledge, compliance, insight into illness, and quality of life after the intervention. Both the short-term and long-term effects observed here are promising. However, the findings need to be confirmed by future research and tested against a suitable control group. Future research also needs to determine whether psychoeducation via film is just as effective as other methods, such as standard group psychoeducation led by an expert or a peer. Psychoeducation via film could be a useful tool in different situations. For instance, it could be used in hospitals that are short of staff. Patients could start the program at the hospital and continue watching the films after discharge, or watch them again later. Relatives would get the chance of receiving information about schizophrenia, even when there are no groups offered for them. In addition, these films could be used to educate new staff. Thus, films could be a versatile addition to the standard treatment, especially in view of scarce resources.

## Abbreviations

ANOVA: Analysis of variance
ANCOVA: Analysis of covariance
CI: Confidence interval
MARS: Medication Adherence Rating Scale
